# A study on the current status of knowledge, attitude, and practice regarding infectious disease prevention and control among outpatient medical staff at a maternity and childcare hospital

**DOI:** 10.1097/MD.0000000000048057

**Published:** 2026-03-27

**Authors:** Shanshan Yang, Huayong Ying, Juping Chen, Caiqun Huang, Hongjuan Tang, Jingjing Xu

**Affiliations:** a Outpatient Office, Jinhua Maternity and Child Health Care Hospital, Jinhua City, Zhejiang Province, China; b Department of Hospital Infection Management, Zhejiang Jinhua Guangfu Tumor Hospital, Jinhua City, Zhejiang Province, China.

**Keywords:** infectious disease, maternity and childcare hospital, medical staff, outpatient, prevention and control knowledge, attitude, and practice, related infection

## Abstract

This paper seeks to probe the current status of the knowledge, attitude, and practice (KAP) of outpatient medical staff regarding the prevention and control of infectious diseases at a maternity and childcare hospital. A survey was done among 168 outpatient medical staff members selected through convenience sampling, with a specifically designed questionnaire adopted to assess their KAP on infectious disease prevention and control. For data analysis, SPSS 23.0 statistical software was harnessed. Based on different types of data, *t* tests or χ^2^ tests were taken for group comparisons. Logistic regression analysis was implemented to identify relevant influencing factors. Among the 168 medical personnel who participated in the survey, 166 valid questionnaires were obtained, yielding an effective rate of 98.81%. The total score for the infectious disease prevention and control KAP questionnaire among the 166 respondents was (69.75 ± 7.88) points, with scores for the knowledge, attitude, and practice dimensions being (20.05 ± 3.22), (24.19 ± 3.55), and (25.51 ± 3.56) points, respectively. Multifactor logistic regression analysis unraveled that having fewer than 10 years of work experience (odds ratio [OR] = 4.289, 95% confidence interval [CI] = 1.267–14.514), education below a bachelor’s degree (OR = 5.859, 95% CI = 1.420–24.169), administrative/technical positions (OR = 5.244, 95% CI = 1.656–16.601), junior and intermediate professional titles (OR = 4.802, 95% CI = 1.333–17.302), and fewer than 3 training sessions (OR = 6.534, 95% CI = 1.375–31.038) were independent risk factors affecting the knowledge of infectious disease prevention and control among outpatient medical staff (*P* < .05). As for independent risk factors influencing practices, they included education below a bachelor’s degree (OR = 4.289, 95% CI = 1.255–14.657), administrative/technical positions (OR = 4.764, 95% CI = 1.319–17.198), junior professional titles (OR = 5.063, 95% CI = 1.202–21.341), fewer than 3 training sessions (OR = 4.894, 95% CI = 1.382–17.326), lack of clinical teaching (OR = 5.590, 95% CI = 1.094–28.552), as well as a moderate level of importance placed on infection control (OR = 5.063, 95% CI = 1.596–16.062; *P* < .05). Overall, outpatient medical staff at the maternity and childcare hospital exhibited notable gaps in their KAP regarding infectious disease prevention and control. Factors such as shorter work experience, lower educational qualifications, nonclinical roles, junior and intermediate professional titles, and fewer training sessions on infectious diseases contributed to these deficiencies.

## 1. Introduction

The knowledge-attitude-practice (KAP) theory is a health promotion framework that emphasizes the progression from learning and accumulating knowledge to forming positive attitudes and beliefs, which then translate into goals and actions.^[1]^ As vital members of healthcare institutions, medical staff are responsible not only for providing high-quality medical services to patients but also for maintaining their own health and safety. This is particularly crucial during periods of high infectious disease prevalence when healthcare workers face elevated infection risks. The KAP levels of medical personnel directly influence the effectiveness of infectious disease prevention and control efforts. Enhancing their awareness and skills in infection prevention and control has become a priority task in healthcare management.^[2,3]^ Maternity and childcare hospitals are unique medical institutions where outpatient medical staff frequently interact with vulnerable groups such as pregnant women and children.^[4]^ With the emergence of new infectious diseases and increasing virus mutations in recent years, the pressure on these medical staff to prevent infections has intensified. They must provide attentive patient care while constantly safeguarding their own health and safety.^[5]^ Thus, it is essential to comprehensively understand the current state of infectious disease prevention and control KAP among this group and to conduct targeted research accordingly.

Research has unveiled that the level of infectious disease prevention knowledge among medical staff directly impacts their practices, subsequently influencing the effectiveness of infection control efforts.^[6]^ Some medical personnel have an inadequate understanding of infectious disease prevention and control, leading to incorrect practices in their work.^[7]^ Additionally, personal protective awareness and behaviors among medical staff are generally weak; improper procedures or a lack of strong protective awareness can culminate in them contracting related diseases. Moreover, the mental health status of medical staff also affects their infection prevention and control practices. Prolonged work-associated stress and anxiety can cause distraction, augmenting the risk of infection.^[8]^ Therefore, it is of particular importance to comprehensively assess the current KAP status of medical personnel regarding infectious disease prevention and control, identify existing problems, and implement targeted interventions.

Implementing relevant research on outpatient medical staff in maternity and childcare hospitals is of significant practical importance. On one hand, this group frequently interacts with vulnerable populations, making it crucial to enhance their awareness and skills in infectious disease prevention to protect the health of pregnant women and children.^[9]^ On the other hand, as a specialized medical institution, the infection prevention and control status of outpatient medical staff in these hospitals may differ from those in general hospitals. Staff members from maternity and childcare hospitals may place greater emphasis on maternal and child healthcare, with potentially different knowledge structures and skill requirements for infection control compared with their counterparts in general hospitals.^[10]^ They face higher risks of infectious disease exposure while being tasked with providing high-quality care to mothers and children. Hence, strengthening the research on the current state of infection prevention and control KAP among outpatient medical staff in maternity and childcare hospitals and implementing targeted interventions are of practical significance to enhancing their infection control capabilities and ensuring maternal and child health.^[11]^ Previous research has typically analyzed the KAP status of hospital medical staff regarding infectious disease prevention and control, with fewer studies focusing specifically on outpatient staff. This study builds on previous research to examine the KAP status of outpatient medical staff at a local maternity and childcare hospital concerning infectious disease prevention and control and explores factors influencing their KAP. The objective is to provide valuable guidance for clinical practice.

## 2. Materials and methods

### 2.1. General data

This study was approved by the Ethics Committee of Zhejiang Jinhua Guangfu tumor Hospital. A survey was conducted involving 168 outpatient medical staff from a maternity and childcare hospital, selected via the convenience sampling method. The study adhered to the ethical guidelines of the Helsinki Declaration.

Inclusion criteria: staff working in the outpatient department of the maternity and childcare hospital; individuals with normal mental status capable of effective communication; voluntary participation in the study.

Exclusion criteria: visiting trainees, normalized training participants, and rotating staff; interns; those with continuous or cumulative leave exceeding 3 days.

This study adopted a census-based convenience sampling approach. At the time of investigation, the total number of outpatient medical staff in the hospital was 168, all of whom met the inclusion criteria and were invited to participate. Therefore, the sample size represented the full accessible population of outpatient medical staff in this institution, ensuring adequate representativeness within the single-center context.

### 2.2. Methods

With the use of the revised “Infectious Disease Prevention and Control KAP Questionnaire for Medical Personnel” by Jiang et al,^[12]^ the study evaluated the current KAP status of medical staff regarding infectious disease prevention and control: Knowledge of prevention and control: This encompasses areas such as infection prevention and control in invasive procedures, preexamination and triage, standard preventive cleaning, disinfection, and sterilization, medical waste disposal, patient isolation, infectious disease monitoring and reporting, and the use of protective equipment. Each area contains 4 items, totaling 32 items. Attitudes and practices towards prevention and control each contain 1 item, totaling 8 items. For knowledge, correct answers score 1 point, and incorrect answers score 0 points. For attitudes, a 5-point Likert scale is employed, where “strongly agree” scores 4 points and “strongly disagree” scores 0 points. For practice, a 5-point Likert scale is used, where “never achieved” scores 1 point and “always achieved” scores 4 points. The maximum score for the questionnaire is 96 points, with higher scores denoting higher KAP levels among medical personnel. The test–retest reliability and validity values for each dimension were 0.932, 0.882, and 0.844, respectively.

### 2.3. Data collection and quality control

The questionnaire content was imported into Wenjuanxing, a survey platform, and a link and QR code were generated. Medical staff could fill out the questionnaire by scanning the code. The survey was conducted anonymously, with each person allowed to respond only once. After the questionnaire was completed, it could be submitted. The survey was coordinated by contacting the head of outpatient medical staff and proceeded through a unified approach. The survey was conducted over a period of 7 consecutive working days, from March 1 to March 7, 2023. During this period, outpatient medical staff voluntarily completed the online questionnaire. After the survey closed, data were exported to an Excel spreadsheet. The expert group reviewed the data for quality, removing any questionnaires that met the following criteria: completion time <2 minutes or responses showing a clear pattern.

### 2.4. Multifactor analysis

General data were harvested from the medical staff, including gender (male/female), age, years of work experience, education level (bachelor’s degree or above, below bachelor’s degree), position (administrative, clinical, technical), professional title (senior, intermediate, junior), number of knowledge training sessions, clinical teaching experience, and level of importance placed on infection control (high, moderate). Logistic regression was utilized to analyze the independent risk factors affecting KAP related to infectious disease prevention and control among outpatient medical staff in the maternity and childcare hospital.

### 2.5. Statistical analysis

SPSS 20.0 software was harnessed for data processing. For unordered categorical data, the χ^2^ test was adopted. Measurement data were represented as ($\bar{x}\pm s$) and analyzed through the *t* test. Multifactor analysis was performed via logistic regression. The significance level for tests was set at α = 0.05.

## 3. Results

### 3.1. Survey results

A total of 168 medical staff participated in the survey, with 166 valid questionnaires harvested, resulting in an effective rate of 98.81%. The total score for the infectious disease prevention and control KAP questionnaire among the 166 respondents attained (69.75 ± 7.88) points, with scores for the knowledge, attitude, and practice dimensions being (20.05 ± 3.22) points, (24.19 ± 3.55) points, and (25.51 ± 3.56) points, respectively. The KAP scores for outpatient medical staff are presented in Table 1.

**Table 1:** Survey results ($\bar{x}\pm \;s$, points).

Items	Knowledge	Attitude	Practice
Infection prevention and control in invasive procedures	2.47 ± 0.26	3.45 ± 0.46	3.66 ± 0.54
Preexamination and triage	2.58 ± 0.31	3.56 ± 0.37	3.71 ± 0.45
Standard preventive cleaning, disinfection, and sterilization	2.61 ± 0.27	3.34 ± 0.65	3.74 ± 0.39
Medical waste disposal	3.04 ± 0.32	3.22 ± 0.38	3.68 ± 0.41
Patient isolation	3.15 ± 0.36	3.58 ± 0.42	3.56 ± 0.53
Infectious disease monitoring and reporting	3.22 ± 0.34	3.77 ± 0.43	3.89 ± 0.46
Use of protective equipment	2.98 ± 0.36	3.27 ± 0.35	3.27 ± 0.45
Total score	20.05 ± 3.22	24.19 ± 3.55	25.51 ± 3.56

### 3.2. Single-factor analysis of factors affecting outpatient medical staff’s KAP on infectious disease prevention and control

General data were compared between the 2 groups, and substantial variances were discovered in the infectious disease prevention and control knowledge scores among medical staff with varying years of work experience, educational levels, job positions, professional titles, and number of training sessions attended (*P* < .05). Additionally, noticeable differences were observed in the attitude scores toward infectious disease prevention and control based on educational levels, job positions, professional titles, number of training sessions, clinical teaching experience, and the importance placed on infection control (*P* < .05). Furthermore, the practice scores associated with infectious disease prevention and control differed significantly by educational levels, job positions, professional titles, number of training sessions, clinical teaching experience, and the emphasis placed on infection control (*P* < .05; see Table 2).

**Table 2:** Single-factor analysis of factors affecting outpatient medical staff’s KAP on infectious disease prevention and control.

General information		n	Knowledge		Attitude		Practice	
			Scores	*t*, *P*		*t*, *P*		*t*, *P*
Gender	Male	87	20.03 ± 2.55	0.089, .929	24.33 ± 2.17	0.828, .409	25.49 ± 4.33	0.609, .952
	Female	79	20.07 ± 3.22		24.04 ± 2.34		25.53 ± 4.11	
Age (yr)	≥30	92	20.06 ± 2.17	0.057, .955	24.23 ± 2.66	0.229, .819	25.52 ± 2.67	0.048, .962
	<30	74	20.04 ± 2.33		24.14 ± 2.33		25.50 ± 2.66	
Work experience (yr)	≥10	82	22.35 ± 2.41	12.368, <.001	24.22 ± 2.67	0.938, .350	25.52 ± 2.61	0.048, .962
	<10	84	17.80 ± 2.33		24.16 ± 2.55		25.50 ± 2.73	
Education level	Bachelor’s degree or above	87	21.98 ± 3.15	8.912, <.001	25.97 ± 3.22	8.404, <.001	27.33 ± 3.76	6.978, <.001
	Below bachelor’s degree	79	17.92 ± 2.67		22.23 ± 2.41		23.51 ± 3.24	
Position	Administrative	54	18.06 ± 4.11	36.065, <.001	22.91 ± 3.23	19.795, <.001	24.33 ± 3.24	13.327, <.001
	Clinical	59	23.45 ± 3.11		26.15 ± 3.22		27.24 ± 3.04	
	Technical	53	18.29 ± 4.25		23.31 ± 2.45		24.79 ± 3.44	
Professional title	Senior	61	23.66 ± 2.71	77.957, <.001	25.33 ± 2.62	9.839, <.001	27.02 ± 3.05	12.510, <.001
	Intermediate	58	17.68 ± 3.45		23.31 ± 2.45		24.65 ± 2.41	
	Junior	47	18.29 ± 2.11		23.80 ± 2.66		24.61 ± 3.44	
Number of training sessions (times)	≥3	89	24.56 ± 2.68	24.787, <.001	26.32 ± 2.76	11.458, <.001	27.55 ± 3.24	10.318, <.001
	<3	77	14.84 ± 2.32		21.73 ± 2.34		23.15 ± 3.14	
Clinical teaching	Yes	92	20.08 ± 3.11	0.158, .874	25.89 ± 2.67	9.108, <.001	27.84 ± 3.02	10.406, <.001
	No	74	20.01 ± 2.44		22.08 ± 2.69		22.61 ± 3.45	
Emphasis placed on infection control	High	80	20.07 ± 2.66	0.106, .916	25.87 ± 2.89	7.414, <.001	26.93 ± 3.12	5.266, <.001
	Moderate	86	20.03 ± 2.19		22.63 ± 2.74		24.19 ± 3.55	

KAP = knowledge, attitude, and practice.

### 3.3. Multivariate analysis of factors influencing outpatient medical staff’s knowledge of infectious disease prevention and control

Variables from Table 2 were assigned values according to the method in Table 3 and input into a logistic regression equation for multivariate logistic regression analysis. As evidenced by the outcomes, having fewer than 10 years of work experience, an education level below a bachelor’s degree, administrative/technical job positions, junior/intermediate professional titles, and fewer than 3 training sessions were considered independent risk factors affecting outpatient medical staff’s knowledge of infectious disease prevention and control (*P* < .05; refer to Tables 3 and 4 for details).

**Table 3:** Assignment method.

Factors	Assignment
Work experience	1 = <10 yr; 0 = ≥10 yr
Education level	1 = below bachelor’s degree; 0 = bachelor’s degree or above
Position	1 = administrative or technical; 0 = clinical
Professional title	1 = junior or intermediate; 0 = senior
Number of training sessions	1 = moderate; 0 = high

**Table 4:** Multivariate analysis of factors influencing outpatient medical staff’s knowledge of infectious disease prevention and control.

Independent variables	β value	SE value	Wald χ^2^ value	OR	95% CI	*P* value
Work experience	1.456	0.622	5.480	4.289	1.267–14.514	.020
Education level	1.768	0.723	5.980	5.859	1.420–24.169	.015
Position	1.657	0.588	7.941	5.244	1.656–16.601	.005
Professional title	1.569	0.654	5.756	4.802	1.333–17.302	.017
Number of training sessions	1.877	0.795	5.574	6.534	1.375–31.038	.019

CI = confidence interval, OR = odds ratio, SE = standard error.

### 3.4. Multivariate analysis of factors influencing outpatient medical staff’s attitudes toward infectious disease prevention and control

Single-factor variables from Table 2 were assigned values based on the method in Table 5 and input into a logistic regression equation for multivariate analysis. It transpired that having an education level below a bachelor’s degree, administrative/technical job positions, junior/intermediate professional titles, fewer than 3 training sessions, no clinical teaching experience, and a moderate emphasis on infection control were regarded as independent risk factors influencing outpatient medical staff’s attitudes toward infectious disease prevention and control (*P* < .05; refer to Tables 5 and 6).

**Table 5:** Assignment method.

Factors	Assignment
Education level	1 = below bachelor’s degree; 0 = bachelor’s degree or above
Position	1 = administrative or technical; 0 = clinical
Professional title	1 = junior or intermediate; 0 = senior
Number of training sessions	1 = <3 times; 0 = ≥3 times
Clinical teaching	1 = no; 0 = yes
Emphasis on infection control	1 = moderate; 0 = high

**Table 6:** Multivariate analysis of factors influencing outpatient medical staff’s attitudes toward infectious disease prevention and control.

Independent variables	β value	SE value	Wald χ^2^ value	OR	95% CI	*P* value
Education level	1.566	0.741	4.466	4.787	1.120–20.458	.035
Position	1.432	0.566	6.401	4.187	1.381–12.697	.012
Professional title	1.679	0.721	5.423	5.360	1.305–22.025	.020
Number of training sessions	1.597	0.644	6.149	4.938	1.398–17.448	.014
Clinical teaching	1.435	0.543	6.984	4.200	1.449–12.174	.009
Emphasis on infection control	1.399	0.562	6.100	4.007	1.332–12.055	.014

CI = confidence interval, OR = odds ratio, SE = standard error.

### 3.5. Multivariate analysis of factors influencing outpatient medical staff’s practices in infectious disease prevention and control

Single-factor variables mentioned in Table 2 were assigned values as per the method in Table 7 and input into a logistic regression equation for multivariate analysis. The outcomes confirmed that having an education level below a bachelor’s degree, administrative/technical job positions, junior/intermediate professional titles, fewer than 3 training sessions, no clinical teaching experience, and a moderate emphasis on infection control were independent risk factors affecting outpatient medical staff’s practices (*P* < .05; see Tables 7 and 8).

**Table 7:** Assignment method.

Factors	Assignment
Education level	1 = below bachelor’s degree; 0 = bachelor’s degree or above
Position	1 = administrative or technical; 0 = clinical
Professional title	1 = junior or intermediate; 0 = senior
Number of training sessions	1 = <3 times; 0 = ≥3 times
Clinical teaching	1 = no; 0 = yes
Emphasis on infection control	1 = moderate; 0 = high

**Table 8:** Multivariate analysis of factors influencing outpatient medical staff’s practices in infectious disease prevention and control.

Independent variables	β value	SE value	Wald χ^2^ value	OR	95% CI	*P* value
Education level	1.456	0.627	5.392	4.289	1.255–14.657	.021
Position	1.561	0.655	5.680	4.764	1.319–17.198	.018
Professional title	1.622	0.734	4.883	5.063	1.202–21.341	.028
Number of training sessions	1.588	0.645	6.062	4.894	1.382–17.326	.014
Clinical teaching	1.721	0.832	4.279	5.590	1.094–28.552	.039
Emphasis on infection control	1.622	0.589	7.584	5.063	1.596–16.062	.006

CI = confidence interval, OR = odds ratio, SE = standard error.

## 4. Discussion

Over the past years, with the rapid development of the socioeconomic landscape, the demand for healthcare services has dramatically increased. As frontline personnel directly providing medical services, the medical staff at maternity and childcare hospitals bear a crucial responsibility in preventing and controlling infectious diseases.^[13]^ Nevertheless, existing studies indicate that these medical workers have notable deficiencies in their knowledge, risk awareness, and preventive practices regarding infectious disease control, which may heighten their risk of contracting infectious diseases and pose potential infection hazards to patients.^[14]^ With an eye to addressing this issue, the present study seeks to comprehensively evaluate the infectious disease prevention and control knowledge, risk perception, and preventive practices of outpatient medical staff at a maternity and childcare hospital through a questionnaire survey, exploring their current status and influencing factors.

During this investigation, it was discovered that the total score of the infectious disease prevention and control KAP questionnaire among the 166 respondents was (69.75 ± 7.88) points, suggesting that the participating outpatient medical staff need improvement in their knowledge, attitudes, and practices in terms of infection control. It is necessary to enhance relevant knowledge and skills in clinical practice. The multivariate logistic regression analysis in this research demonstrated that having fewer than 10 years of work experience, possessing an education level below a bachelor’s degree, holding administrative/technical positions, having junior or intermediate professional titles, and receiving fewer than 3 training sessions on infection control were independent risk factors affecting the infection prevention and control knowledge of outpatient medical staff. Medical staff with shorter work experience may lack practical experience and the infection control knowledge and skills accumulated over time. In contrast, those with longer work experience have amassed more relevant knowledge through practice and training.^[15]^ Medical personnel with lower educational attainment may have deficiencies in foundational medical knowledge and key theoretical understanding. Educational level was found to be a significant factor influencing KAP, which may be attributed to differences in systematic medical training and critical thinking abilities. Medical staff with higher educational attainment are more likely to have received structured education on infection prevention and evidence-based practice, enabling better comprehension and application of infection control guidelines. Similar findings have been reported in studies conducted in China and other countries, where higher education was consistently associated with better infection prevention knowledge and practices.^[16-19]^

The regression analysis implemented in this study identified several independent risk factors affecting the infection control attitudes of outpatient medical staff: educational attainment below a bachelor’s degree, holding administrative/technical positions, having junior or intermediate professional titles, fewer than 3 training sessions on infection control, lack of clinical teaching experience, and a moderate level of concern for infection control. A lower educational background may denote that medical staff have a weaker grasp of infection control knowledge and lack systematic training and learning, which can affect their attentiveness and execution of infection control measures.^[20]^ Administrative and technical positions usually do not involve direct clinical work, and these staff members lack frontline clinical experience. As opposed to clinicians, they might not fully appreciate the importance of infection control.^[21]^ Junior or intermediate professional titles signify relatively less work experience and weaker professional skills, which might contribute to lower emphasis and effectiveness in infection control.^[22]^ Insufficient systematic training on infection control could result in an inadequate understanding of the importance of infection control measures.^[23]^ Lack of frontline clinical teaching experience may cause poor mastery of practical operations and a weaker ability to implement infection control measures.^[24]^ If medical staff do not fully recognize the significance of infection control, it will be difficult for them to develop good infection control habits in their daily work.^[25]^

Our regression analysis uncovered several independent risk factors influencing the infection control practices of outpatient medical staff: educational level below a bachelor’s degree, holding administrative/technical positions, having junior or intermediate professional titles, fewer than 3 training sessions on infection control, lack of clinical teaching experience, and a moderate level of concern for infection control. Medical staff with lower educational levels may fail to develop a deep understanding and appreciation of infection prevention and control, which can impact their ability to adopt effective infection control practices.^[26]^ Those in administrative and technical roles may have less practical experience compared with frontline clinicians, leading to a weaker emphasis on and skill in infection control.^[27]^ Staff with lower professional titles may be short of extensive clinical experience and specialized knowledge, impacting their ability to take appropriate infection control measures.^[28]^ Insufficient training on infection prevention and control can result in inadequate knowledge and skills, preventing the effective implementation of related measures.^[29]^ The absence of frontline clinical teaching and guidance might hinder medical staff from promptly assimilating and applying the latest infection control knowledge and skills.^[30]^ If medical staff do not recognize the importance of infection control, their practices may also be negatively affected.^[31]^

This study has several limitations. First, due to its cross-sectional design, causal relationships between influencing factors and KAP could not be established. Second, convenience sampling may have introduced selection bias, limiting the representativeness of the sample. Third, as this was a single-center study conducted in a maternity and childcare hospital, the findings may not be fully generalizable to other healthcare settings or regions. Future multicenter and longitudinal studies with larger sample sizes are warranted to validate and expand upon these findings. This study adopted a convenience sampling method, which may have introduced selection bias. Medical staff who were more interested in infection prevention and control or more motivated to participate in surveys may have been more likely to respond, potentially leading to an overestimation of KAP levels.

To sum up, shorter job tenure, education below a bachelor’s degree, administrative/technical roles, junior or intermediate professional titles, insufficient participation in training sessions, lack of clinical teaching experience, and a moderate level of concern for infection prevention and control all influence the infectious disease prevention and control KAP of outpatient medical staff in maternity and childcare hospitals. Hence, in clinical settings, enhancing educational qualifications, strengthening knowledge training, optimizing work distribution, and improving teaching mechanisms could help improve the infection prevention and control KAP of medical staff.

## Author contributions

**Conceptualization:** Shanshan Yang, Huayong Ying, Juping Chen, Caiqun Huang, Hongjuan Tang, Jingjing Xu.

**Data curation:** Shanshan Yang, Huayong Ying, Juping Chen, Caiqun Huang, Hongjuan Tang, Jingjing Xu.

**Formal analysis:** Shanshan Yang, Huayong Ying, Juping Chen, Caiqun Huang, Hongjuan Tang, Jingjing Xu.

**Funding acquisition:** Shanshan Yang, Huayong Ying.

**Investigation:** Shanshan Yang, Huayong Ying.

**Writing – original draft:** Shanshan Yang, Huayong Ying, Hongjuan Tang.

**Writing – review & editing:** Shanshan Yang, Huayong Ying, Hongjuan Tang.
